# Significant Biotransformation of Arsenobetaine into Inorganic Arsenic in Mice

**DOI:** 10.3390/toxics11020091

**Published:** 2023-01-18

**Authors:** Jichao Zhang, Zijun Ye, Liping Huang, Qianyu Zhao, Kaige Dong, Wei Zhang

**Affiliations:** School of Environmental Science and Engineering, Guangzhou University, Guangzhou 510006, China

**Keywords:** arsenobetaine, arsenate, biodegradation, bioaccumulation, mouse

## Abstract

Arsenic (As) is extremely toxic to living organisms at high concentrations. Arsenobetaine (AsB), confirmed to be a non-toxic form, is the main contributor to As in the muscle tissue of marine fish. However, few studies have investigated the biotransformation and biodegradation of AsB in mammals. In the current study, C57BL/6J mice were fed four different diets, namely, Yangjiang and Zhanjiang fish diets spiked with marine fish muscle containing AsB, and arsenite (As(III)) and arsenate (As(V)) diets spiked with As(III) and As(V), respectively, to investigate the biotransformation and bioaccumulation of AsB in mouse tissues for 42 d. Different diets exhibited different As species distributions, which contributed to varying levels of As bioaccumulation in different tissues. The intestines accumulated the highest level of As, regardless of form, which played a major part in As absorption and distribution in mice. We observed a significant biotransformation of AsB to As(V) following its diet exposure, and the liver, lungs, and spleen of AsB-treated mice showed higher As accumulation levels than those of As(III)- or As(V)-treated mice. Inorganic As showed relatively high accumulation levels in the lungs and spleen after long-term exposure to AsB. Overall, these findings provided strong evidence that AsB undergoes biotransformation to As(V) in mammals, indicating the potential health risk associated with long-term AsB intake in mammals.

## 1. Introduction

As a common toxic substance, arsenic (As) ranks first on the priority list of hazardous substances published by the U.S. Environmental Protection Agency [1]. Owing to significant industrial and agricultural development, a large number of toxic pollutants such as arsenite (As(III)) and arsenate (As(V)) have been discharged into the ocean [2,3]. Generally, inorganic As (As(III) and As(V)) is more toxic than methylated pentavalent arsenicals such as monomethylarsonate (MMA(V)) and dimethylarsinate (DMA(V)), which are more toxic than arsenobetaine (AsB). Reportedly, AsB and arsenocholine (AsC) are considered the least toxic forms of As [4].

The absorption of As by marine organisms is followed by a series of biotransformation processes [5,6]. Previous studies have found that As concentrations in marine fish (1–10 μg/g) are much higher than those in freshwater fish (<1 μg/g) [7,8]. Moreover, As concentrations in many marine fish vastly exceeded those in most terrestrial foods [9,10,11], with AsB being the final storage species that accumulates in the muscle of marine fish [12,13]. Currently, it is estimated that the concentration of total As (AsB > 90%) in seafood could be 200 times higher than the limit for inorganic As (50 ng/g) in food in many countries [14]. Furthermore, marine fish, as As-contaminated foods, are the predominant source of As in humans. However, whether such high concentrations of AsB pose a potential health hazard to humans has not been clearly determined; therefore, the potential health risk from AsB in marine fish needs to be established.

Humans may become exposed to As via food, leading to physiological dysfunction and the progression of various diseases, such as cardiovascular disease, diabetes, and multiorgan cancers [15]. Furthermore, the biotransformation of inorganic As has received considerable attention owing to its high toxicity and carcinogenic potential [16]. Ingested inorganic As, including As(V) and As(III), could be metabolized via methylation to form MMA and DMA as the end metabolites, which are then excreted via urine in mammals. However, the reactive intermediates of these methylated metabolites, namely monomethylarsonous (MMA(Ⅲ)) and dimethylarsinous (MMA(Ⅲ)) acids, which are more toxic than inorganic As, have been detected in mammalian urine [17,18]. Additionally, the biotransformation of monomethylated and dimethylated As has received considerable attention. MMA(III) can be methylated to DMA(V) and demethylated to As(V) in the presence of mouse cecum homogenate [19]. It has also been observed that ingested MMA(III) is more extensively metabolized to dimethylated As than MMA(V) in mice [20]. Several mechanisms for the biotransformation of dimethylated As have also been proposed. Some studies have shown that DMA(V) is not demethylated and excreted as the major metabolite in the urine of humans and animals, including mice, rats, hamsters, and rabbits [21,22]. In contrast, DMA(V) can be reduced to dimethylarsinous acid (DMA(III)), which can be effectively taken up by the red blood cells of rats, or demethylated to MMA(V) and inorganic As, which are subsequently excreted in the urine of DMA(V)-treated rats [23,24]. Additionally, a previous study showed that 4−15% of orally administered DMA(V) is excreted as trimethylarsenic oxide (TMAO) in the urine of mice [22]. Similarly, another study revealed that rats exposed to drinking water containing DMA(V) for 14 d excreted feces containing TMAO. Moreover, unknown As metabolites have been observed in the urine, feces, and liver of rats and mice exposed to DMA(V) or DMA(III) [25,26].

Seafood, especially marine fish and shellfish, contains high concentrations of AsB, making it an effective route for AsB intake by humans [27,28]. In view of the high concentration of AsB in marine fish, previous studies have examined the bioaccumulation and metabolism of AsB in different mammals. AsB biotransformation, retention, and distribution were investigated in rabbits, rats, and mice, and 98.5% of the administered dose was found to be excreted in 2 d, after which no other arsenicals were detected [29]. Thus, AsB has long been considered nonmetabolizable in rodents, as no other arsenicals and only AsB were observed in the urine of rabbits, rats, or mice orally administered AsB after short-term exposure. When administered orally, AsB is thought to be mainly excreted in the urine, considering the elimination of a fraction of the administered dose within a short time without being metabolized, likely due to its highly polar and hydrophilic characteristics, and is therefore less likely to be retained in vivo [30,31]. However, recent evidence suggests that AsB can be transformed by microorganisms in the human gastrointestinal (GI) tract, with DMA and TMAO as the main degradation products [32]. Similarly, other forms of As, including TMAO, MMA, DMA, tetramethylarsonium, and inorganic As, have been observed in the urine of rats fed AsB-containing diets [33]. However, some other studies reported that AsB may participate in metabolic processes in vivo [32,33,34]. Thus, the distribution and metabolic mechanism of AsB in mammals remains controversial. Additionally, no study has focused on including fish muscle in mouse diets to simulate the biotransformation process. Therefore, it is necessary to further investigate the biotransformation/biodegradation of ingested marine fish muscle containing different As species, especially AsB, in mammals. Further, studies on AsB degradation to inorganic As could contribute to enhancing the understanding of the potential food toxicity and health risks associated with AsB.

Therefore, this study focused on a typical food chain (marine goby fish *Trypauchen vagina*–C57BL/6J mice). Marine benthic goby fish were selected mainly because they naturally contain high As concentrations in marine environments [2]. Total As and As species concentrations in different mouse tissues were specifically examined to analyze the biotransformation process during the transfer from marine fish to mice. Despite its importance, little information is available on the biodegradation of AsB from marine fish muscle in different mammalian tissues. Therefore, the assessment of AsB health hazards derived from marine fish is critical and could provide a theoretical basis for human health risk assessments.

## 2. Materials and Methods

### 2.1. Chemicals and Reagents

Standards as well as extraction and mobile phase solutions were prepared using Milli-Q water (18.2 MΩ cm, IQ 7000, Millipore SAS, Molsheim, France). Further, standard solutions of total As were prepared via serial dilution of 10 mg/L stock solution (Multi-element Calibration standard 2A, Agilent, Palo Alto, CA, USA), while the AsB, As(III), and As(V) solutions were prepared using AsB (C_5_H_11_AsO_2_, Sigma-Aldrich, St. Louis, MO, USA), sodium arsenite (NaAsO_2_, Sigma-Aldrich), and sodium arsenate dibasic heptahydrate (Na_2_HAsO_4_·7H_2_O, Sigma-Aldrich), respectively. Furthermore, the experimental mice were anesthetized using pentobarbital sodium (C_11_H_17_O_3_N_2_Na, biological reagent, Shanghai Yuanye Bio-Technology Co., Ltd., Shanghai, China), and samples collected from the test animals were digested using 65% nitric acid (Merck, Darmstadt, Germany). Additionally, for high-performance liquid chromatography (HPLC), the chemicals used were sodium hydroxide (NaOH, analytical reagent (AR) grade, Macklin, Guangzhou, China), the mobile phase (citric acid (C_6_H_8_O_7_, AR grade)), and sodium 1-hexanesulfonate (C_6_H_13_O_3_S·Na, 98%), which were purchased from Shanghai Chemical Industry Park (Shanghai, China). The different As solutions and extracts were saved in high-density polypropylene centrifuge tubes and stored at 4 °C.

### 2.2. Exposure Diets and Experimental Design

Marine goby fish *T. vagina* (50 kg, 1 year) were collected from the market of Yangjiang (YJ) and Zhanjiang (ZJ), Guangdong Province, China, in July 2019. The marine goby fish *T. vagina* were dissected to obtain muscle tissues, which were then freeze-dried, weighed, and added to basic feed materials for mice to prepare a special mouse diet containing AsB derived from marine fish from a different city using a feed machine. Moreover, 1 mL of As(III) and As(V) solutions were added to 2.5 kg of artificial mouse diets, which served as the As(III) and As(V) diets, respectively.

Five-week-old C57BL/6J mice were chosen for our experiment, which were purchased from the Institute of Laboratory Animal Science, Chinese Academy of Medical Sciences, Beijing, China. The study was designed and reported in accordance with ARRIVE guidelines accessed on 21 October 2019 (https://arriveguidelines.org). Further, all animal experiments were conducted in accordance with the National Institutes of Health Guidelines in the Guidelines for the Care and Use of Laboratory Animals (8th edition). All animal experiments were approved by the Animal Care and Use Committee of Guangzhou University. The experimental environment was kept at 22 ± 1 °C, a light/dark cycle of 12 h/12 h, a relative humidity of 50–60%, and food and water were provided ad libitum. The mice were acclimated to these test conditions for 7 d before starting the exposure experiments. The bedding material and feed were purchased from Guangzhou Saibonuo Biotechnology Co., Ltd. (Guangzhou, China).

After acclimation for 7 d, 50 mice were randomly divided into 5 treatments. Each treatment consisted of two replicates; each cage contained five mice. The mice were provided sterilized deionized water and diets supplemented with goby fish muscle from YJ and ZJ along with As(III) and As(V) solutions each day. The same quantity of food (2.5 kg) was fed every 2 d. The mice were maintained under laboratory conditions for 42 d. The body weight of the mice was measured, and feces were collected daily. During the exposure period, feed and sterilized purified water were supplied normally. The stomach (rinsed to remove food contents residue), intestine (rinsed to remove food content residue), heart, liver, spleen, lung, kidney, and muscle were collected and homogenized for the total As and As forms.

The body weights of the mice were determined daily. Further, fecal samples were collected every day, and the mice were fasted for 24 h, with only drinking water provided, to ensure that their stomachs were empty before the necropsy. On the second day, the mice were narcotized using 0.5% sodium pentobarbital at a dose of 50 mg kg^−1^, and whole-body perfusion was conducted to remove blood from the circulatory system and the mouse organs [35]. Blood samples (ca. 1 mL) were collected for As species analysis at the beginning of the perfusion process. Furthermore, urine and chyme samples were collected from the bladder and small intestine, respectively, for As species distribution analysis, while internal tissues, including the heart, liver, spleen, lungs, kidneys, intestine (rinsed to remove food contents residue), stomach (rinsed to remove food contents residue), and muscle, as well as fecal samples, were collected and homogenized for total analyses of As and As forms.

### 2.3. Total Arsenic Determination

The total concentration of As was analyzed as depicted in an earlier study [6]. The tissues and diets of mice were placed in a freeze-drying machine for 48 h till a constant weight was achieved and then ground into powder to ensure homogenization. About 0.1–0.3 g of samples was placed in 15 mL centrifuge tubes (Corning, Guangzhou, USA), followed by 1 mL HNO_3_. Before placing the samples in a metal bath (MK200-4, Allsheng, China), the tubes were incubated at 25–26 °C for 1 h, allowed to react at 80 °C for 24 h until clarification, and then diluted for detection.

The total As content was detected using inductively coupled plasma mass spectrometry (ICP-MS, Agilent 7900, Japan). Total As bioaccumulation (μg g^−1^) = total As concentrations detected by ICP-MS (μg L^−1^) × solution volume (L)/sample weight (g). Tuna fish standard reference material (SRM) (BCR-627, Institute for Reference Materials and Measurements, Geel, Belgium) was analyzed to evaluate the accuracy of our digestion method. The SRM contained a total As the content of 4.7 ± 0.8 μg g^−1^ (97.9% recovery of 4.8 ± 0.3 μg g^−1^ certified value, n = 6).

### 2.4. Arsenic Speciation Analysis

The As speciation analysis has been modified as previously mentioned [6,11,36]. Approximately 0.1–0.3 g of samples was placed in a microwave digestion system, followed by 8 mL of 1% HNO_3_, and then digested at 100 °C for 1.5 h. Afterward, the reactant was transferred to 15 mL centrifuge tubes and placed in a block at 80 °C for 24 h. Subsequently, Milli-Q water was added multiple times until the reactant was concentrated to 5 mL. Then, 0.22 µm acidified nitrocellulose filters were used to filter the cooling solutions into 2 mL brown sample bottles (Agilent). The separation of As species in the solution was achieved on an Agilent Zobarx SB-Aq column with 20 mM citric acid (C_6_H_8_O_7_) and 5 mM sodium 1-hexanesulfonate (C_6_H_13_O_3_S·Na) as the mobile phase (pH 4.3) using HPLC (Agilent 1260 Infinity II, Germany) with ICP-MS detection. BCR-627 were used to evaluate the accuracy of As species, which contained 3.76 ± 0.14 μg g^−1^ AsB (96.4% recovery, n = 6) and 1.35 ± 0.15 μg g^−1^ DMA (90.0% recovery, n = 6). Spikes were used to confirm the recovery of As(III), As(V), and MMA, which were 87−91%, 88−96%, and 90−96%, respectively.

Two different methods to extract As from feed (adding fish) were analyzed. For the first method, 50% methanol solution was used to extract As via homogenization [6]. The other method has been described above. The results obtained following both extraction methods did not display any AsB degradation (Figure S1). BCR-627 was analyzed to evaluate the accuracy of our digestion method, which showed the following results: 3.71 ± 0.17 μg/g AsB (95.1% recovery, n = 6) and 1.28 ± 0.19 μg/g DMA (85.3% recovery, n = 6). The recoveries of the other three types of As, i.e., As(III), As(V), and MMA, were confirmed with peak values of 82−90, 89−94, and 87−93%, respectively. Further, column recovery was calculated as the ratio of the sums of the contents of different As forms to the total As content determined with the extracted solution [37]. Thus, the recovery rate varied in the range of 89.1–180% (Tables S1 and S2).

### 2.5. Statistical Analysis

R Studio was used for data visualization. Statistical analysis was performed using SigmaPlot 14.0. Linear fitting, nonlinear fitting, and correlation analyses were used to assess the correlations between the data. A probability level (*p*-value) of less than 0.05 was considered statistically significant.

## 3. Results and Discussion

### 3.1. Total Arsenic Content and Arsenic Speciation in Diets

**Figure 1:**
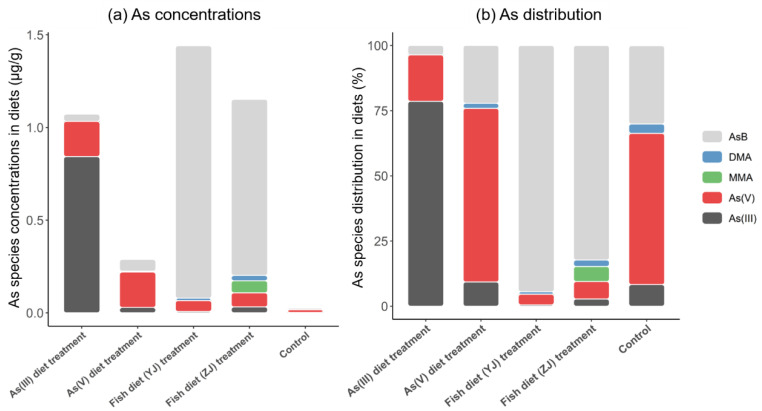
Arsenic species concentrations (µg/g) and distribution (%) in As(III) and As(V) diets, Yangjiang (YJ) and Zhanjiang (ZJ) fish diets, and control diet. Data are represented as the mean ± SD (n = 10).

Figure 1 and Table S2 show the total concentrations and distributions of As and As forms in different diets. Different mouse diets contained different proportions of organic As compounds (MMA, DMA, and AsB) and inorganic As (As(III) and As(V)) (Figure 1). The total As concentrations in the As(III), As(V), fish (YJ), fish (ZJ), and control diets were 1.09, 0.301, 1.44, 1.26, and 0.0289 μg/g, respectively (Table S2). In As(III) diets, As(III) (78.6%) was the predominant As form, followed by As(V) (17.8%), while the percentage of AsB (3.64%) was very low. In As(V) diets, As(V) (66.6%) was the major species, followed by AsB (22.2%), As(III) (9.29%), and DMA (1.94%). In fish diets (YJ), AsB (94.5%) was the primary form of As, followed by As(V) (4.10%). Similarly, in the fish diet (ZJ), AsB (82.3%) was the predominant As species, and MMA (5.76%) was also detected. In control diets, As(V) (58.0%) was the predominant As form, followed by AsB (30.0%), As(III) (8.29%), and DMA (3.63%), which occupied only a small proportion (Table S2). Therefore, inorganic As was the major As species in As(III) and As(V) diets, whereas AsB was the major form in fish (YJ) and fish (ZJ) diets. Overall, the distribution of As in different diets was different.

### 3.2. Differential Bioaccumulation in Various Internal Organs

**Figure 2:**
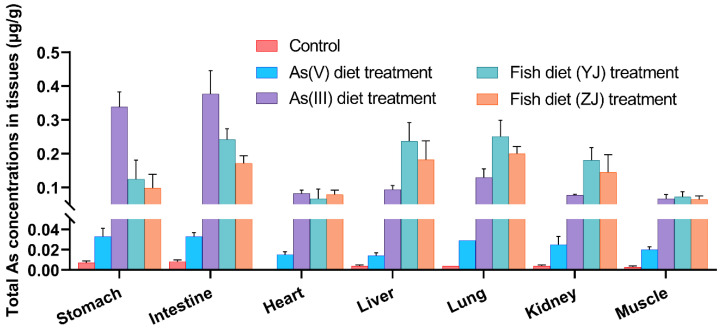
Total As concentrations (µg/g) in the stomach, intestine, heart, liver, lung, kidney, and muscle tissues in mice after As(V) and As(III) diet treatment, Fish diet (YJ) and (ZJ) treatment [Yangjiang (YJ) and Zhanjiang (ZJ) fish diet], and control (control diet exposure). Data are represented as the mean ± SD (n = 10).

The concentrations of total As in major internal tissues, including the stomach, intestine, heart, liver, lung, kidney, and muscle, are presented in Figure 2 and Table S1. The total As concentrations in tissues after the consumption of As(III) and As(V) diets and fish diets were considerably higher than the background concentrations (control diet exposure) (Figure 2), suggesting that the bioaccumulation of As in mice was significantly higher after exposure to food containing As. The range of total As contents was 0.0667–0.377 µg/g after As(III) diet exposure, while the total As concentration was 0.0139–0.0332 µg/g after As(V) diet exposure. Although the difference in the concentrations in these inorganic As diet exposure treatments was evidently due to the different As concentrations in food, the bioaccumulation trend was similar. The highest concentration of total As accumulated in the intestine and stomach after As(III) and As(V) diet exposure (Figure 2). One possible explanation was that most of the inorganic As ingested through the diets was efficiently absorbed by the GI tract [38,39]. Inorganic As damages the gastric mucosal barrier, leading to inflammation, tissue damage, and dysplasia of the gastric gland epithelium, and even enhances the invasion and metastasis of cancer cells by producing reactive oxygen species [40]. This result may explain why inorganic As exposure increases the risk of gastric cancer. Following the consumption of fish diets (YJ and ZJ), the ranges of total As contents were 0.067–0.251 µg/g and 0.065–0.200 µg/g, respectively. Notably, relatively high total As concentrations were observed in the intestine, liver, lung, and kidney, and a relatively low accumulation of total As was observed in the heart, muscle, and spleen after exposure to fish diets (YJ and ZJ). Following control exposure, the total As concentrations in the stomach (0.007 µg/g) and intestine (0.008 µg/g) were higher than those in other tissues (Figure 2). Therefore, diets with different As species contributed to different As bioaccumulation levels in different tissues. The intestine, instead of the stomach, had the capacity to accumulate the highest level of As, regardless of the As forms; thus, the intestine played a significant role in the absorption and distribution of As in mice. Previous studies reported that host organ damage and disease were related to intestinal microbe changes [41].

Specifically, the ingestion of As(III) diet resulted in a relatively high total As concentration in the stomach and intestine (Figure 2). This could be attributed to a strong binding ability between As(III) and SH groups in the body, which occurs during acute As(III) toxicity [42]. The increased tissue accumulation of inorganic As indicated that the inorganic forms were less readily excreted [30]. Interestingly, mice exposed to AsB showed higher total As concentration than those exposed to As(V) and As(III) in the liver, lung, and kidney. Moreover, AsB and inorganic As exhibited differential organ accumulation and tissue localization. Therefore, further studies are necessary to elucidate the specific target organs of AsB in mice.

### 3.3. Arsenic Species Bioaccumulation and Biotransformation in Mice

**Figure 3:**
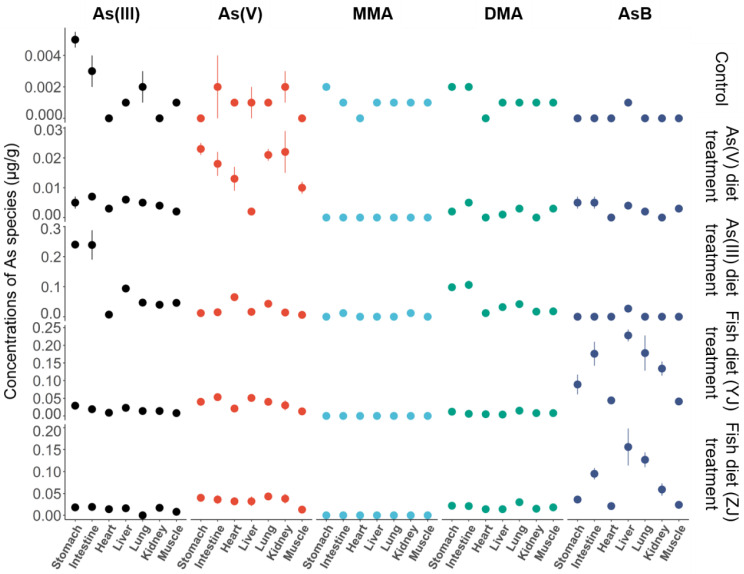
Arsenic species concentrations (µg/g) in the stomach, intestine, heart, liver, lung, kidney, and muscle tissues in mice after As(III) and As(V) diet, Yangjiang (YJ) and Zhanjiang (ZJ) fish diet, and control diet exposure. Data are represented as the mean ± SD (n = 10). The line represents the standard deviation.

**Figure 4:**
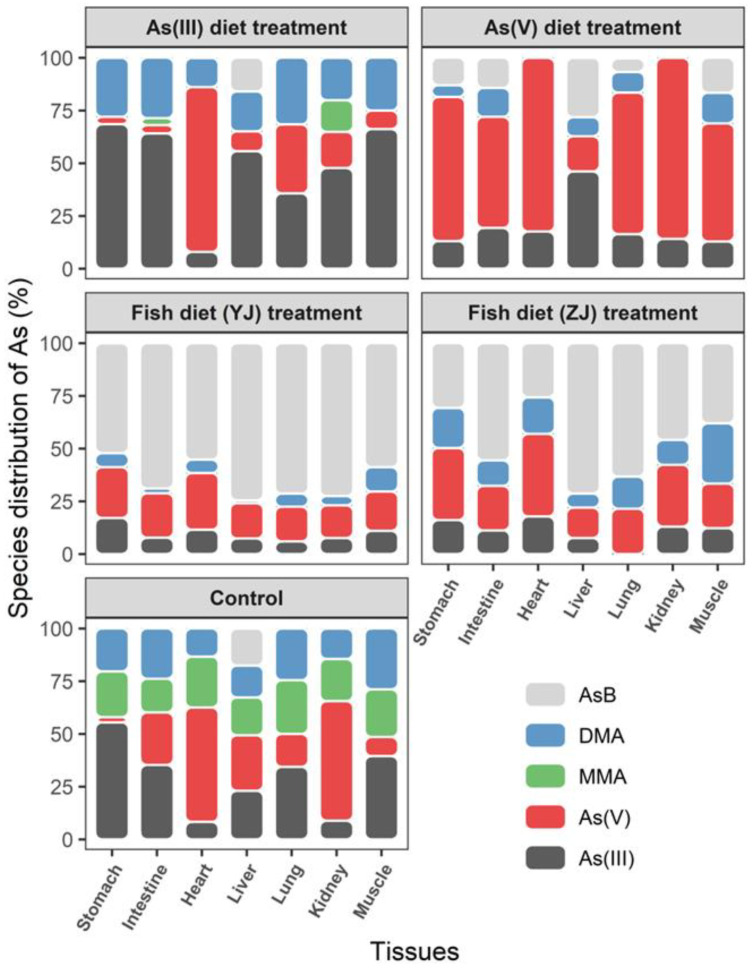
The proportion (%) of different As species in the stomach, intestine, heart, liver, lung, kidney, and muscle tissues in mice after As(III) and As(V) diet, Yangjiang (YJ) and Zhanjiang (ZJ) fish diet, and control diet exposure. Data are represented as the mean ± SD (n = 10).

The concentrations and distributions of As species in different mice tissues are shown in Figure 3 and Figure 4. After As(III) diet exposure, the As(III) concentration was relatively higher than that of other As species (Figure 3). As(III) accounted for the largest proportion in all major tissues except the heart, followed by DMA (Figure 4). As(V) was the major As species in the heart after As(III) diet exposure. One possible explanation is that As(III) was transformed into less toxic As(V) in the heart, which might be a physiological adaptation in mammals [43]. MMA was only detected in the intestines and kidneys after As(III) diet exposure, consistent with previous studies, as MMA is the predominant metabolite in the kidneys [38,44,45]. Kidneys absorb MMA from the blood, and MMA subsequently binds to macromolecules [38,43,46]. After As(V) diet exposure, As(V) represented the largest proportion in all major tissues except the liver, followed by As(III), AsB, and DMA. Thus, As(III) and As(V) accounted for the largest proportions after As(III) diet and As(V) diet exposure, respectively. After entering the blood circulation, inorganic As combines with hemoglobin in plasma and red blood cells is transported to cells through transporters, and then accumulates in the kidneys, lungs, and heart of humans [27].

DMA was detected in all major tissues after As(III) and As(V) diet exposure due to the transformation of inorganic As or MMA. MMA was observed in the intestine but was not in detected diets, indicating that inorganic As was methylated to MMA in the intestine [47]. Inorganic As is efficiently absorbed and metabolized by the reduction of As(V) to As(III), methylated to MMA, and then further methylated to DMA in mice [47,48,49]. DMA and MMA are the major terminal products of As metabolism in mammals [17,30]. Methylation reactions have traditionally been considered a detoxification mechanism because the reactivity and acute toxicity of methylation metabolites to tissue components are lower than those of inorganic compounds [50]. Meanwhile, AsB was also found in the As(III) and As(V) diet exposure treatments (Figure 3 and Figure 4). Therefore, inorganic As accounts for a large proportion in tissues after As(III) and As(V) diet exposure. The transformation of As(III) and As(V) is reversible, followed by sequential methylation to produce MMA and DMA, and AsB is also synthesized.

In this study, after fish diet (YJ and ZJ) exposure, different As species presented similar distributions in the mouse tissues (Figure 3 and Figure 4). AsB was the major As species detected in the liver, intestine, kidney, and lung tissues, followed by As(V). Additionally, As(III) and DMA were present in most organs (Figure 3 and Figure 4). Notably, the As(V) concentration increased obviously in the different tissues after fish diets (YJ and ZJ) were fed. The As(V) concentrations detected in the intestine, stomach, liver, and lung of the group fed fish diets (YJ and ZJ) were relatively higher than those in the As(V) diet and control diet treatment groups (Figure 3 and Table S1).

The amount of AsB consumed in 42 d after fish diet (YJ) exposure was calculated as: 5.95 g (the amount of food each mouse ate one day) × 1.36 μg/g (AsB concentration in fish added diets) × 42 d after fish diet (YJ) exposure = 340 μg, indicated by mass balance calculation. Previous studies pointed out that the AsB retention rate is generally between 0 and 25% in mammals, with variation existing among different species [29,51]. Compared with marine animals, the retention ability of AsB is poor in terrestrial animals [52]. The assimilation efficiency (AE) of AsB was 8 ± 1% and 15 ± 1% in Atlantic salmon and Atlantic cod, respectively. The mean AE of dietary As(V) in marine juvenile fish was 5.5% [53]. Thus, 10% was the AE value in this study: 340 μg × 10% /24.5 g (exposed mice weight) = 1.39 μg/g. The total AsB concentration was 1.26 μg/g in all tissue samples (Table S1), which was less than the AsB concentrations ingested from feed, which indicated that AsB in tissues had been degraded to other As species. The consumed As(V) amount was calculated as follows: {5.95 g (the amount of food each mouse ate one day) × 0.059 μg/g [As(V) concentration in fish added diets] × 42 d = 14.7 μg, 14.7 μg × 10%/24.5 g (exposed mice weight) = 0.0600 μg/g}. The sum of the As(V) concentration was 0.249 μg/g in all tissue samples (Table S1), which was higher than the ingested As(V) concentration in the AsB fish treatment.

As above, the amount of AsB consumed in 42 d after fish diet (ZJ) exposure was calculated as: 5.95 g (the amount of food each mouse ate one day) × 0.95 μg/g (AsB concentration in fish-added diets) × 42 d after fish diet (ZJ) exposure = 237 μg indicated mass balance calculation. We chose the AE as 10%, which is 237 μg × 10% /24.5 g (mean weight of mice) = 0.967 μg/g. The total concentration of AsB was 0.487 μg/g in all tissue samples (Table S1), which was less than the AsB concentrations ingested from feed, which indicated that AsB in tissues had been degraded to other As species; the total As(V) concentrations were calculated as follows: 5.95 g (the amount of food each mouse ate one day) × 0.0762 μg/g (As(V) concentration in fish added diets) × 42 d = 19.0 μg, 19.0 μg × 10%/24.5 g (weight of exposed mice) = 0.0776 μg/g. The As(V) concentration was 0.234 μg/g in all tissue samples in the AsB fish treatment (Table S1), which was obviously higher than the As(V) ingested from the fish-added feed. These results suggested that As(V) could be biodegraded by AsB.

Although the biodistribution, biotransformation, and toxicity of inorganic As in mammals have been well studied, the bioaccumulation and biotransformation of AsB are still largely unknown [33,38,39,54]. Previous studies suggested that orally administered AsB is completely absorbed in the GI tract and excreted unchanged via the urine; it is not metabolized in mice, rats, and rabbits [29,42]. AsB ingested from food is efficiently absorbed by the GI tract and accumulates in humans [55], and AsB displays higher bioaccumulation in the intestine, lung, kidney, and liver in humans [56]. In this study, AsB accumulated in the different tissues of mice instead of being excreted completely unchanged via the urine. Meanwhile, As(V) also was detected at a relatively higher concentration and percentage, which may be a potential health risk in mice. However, the mechanisms of AsB degradation in mouse tissues require further study in the future.

### 3.4. Potential Toxicological Risks of Different Arsenic Forms

**Figure 5:**
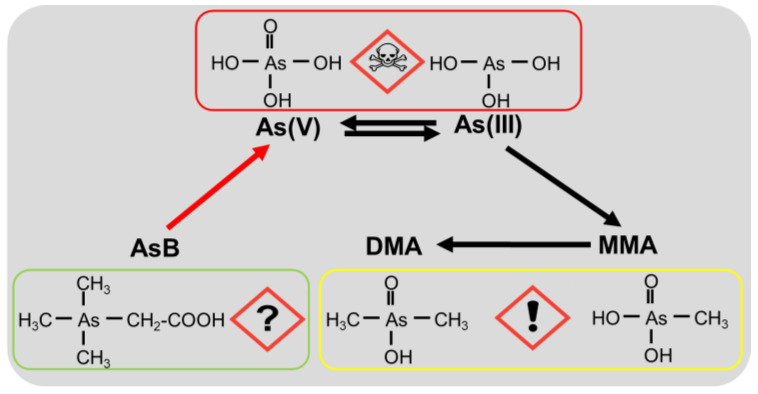
Arsenobetaine (AsB) ingested from seafood transformed to As(V) in tissues.

The toxicity of ingested As largely depends on the amount of accumulated inorganic As in different organs. Several arsenicals have been classified into different groups by the International Agency for Research on Cancer (IARC) based on their toxicity and carcinogenicity in humans. In particular, inorganic As, including As(III) and As(V), are classified as highly carcinogenic to humans (Group 1), whereas AsB is considered non-toxic (Group 3), despite its carcinogenicity in humans (Figure 5). A similar toxicity classification could also be concluded based on the acute toxicity of As species. For instance, the LD50 of inorganic As(III) is approximately 200-fold that of AsB; moreover, AsB has a higher LD_50_ value than inorganic As for acute oral exposure [57]. In this study, AsB was predominantly biotransformed into inorganic As, presumably by intestinal microorganisms. Moreover, the acute toxicity of ingested As is not a proper indicator of its potential health risk given that, in reality, it is more likely for individuals to be exposed to As at considerably lower levels but for a relatively longer duration. AsB, the most prevalent form of As in seafood, has long been considered non-toxic and nonmetabolizable in mammals, and its chronic toxicity has rarely been evaluated [58]. However, the results of this research showed that AsB can be readily degraded to other forms of As, especially As(V) in some tissues. Furthermore, we observed that AsB-treated mice had a higher level of accumulated total As in their intestine, liver, lung, and kidney compared with inorganic As-treated mice. Additionally, AsB-treated mice showed higher levels of inorganic As in their internal tissues than control mice and As(V)-diet mice under 42 d exposure, indicating that the health risk of long-term AsB intake from seafood.

## 4. Conclusions

The potential health risk of As is not only based on the type of As species but also on its biotransformation in vivo. Besides occupational or contaminated drinking-water exposure, seafood consumption is another major source of As intake, especially AsB. Studies on the biotransformation of ingested AsB could considerably improve our understanding of the As potential health risks. Therefore, we analyzed the bioaccumulation and biotransformation of AsB and inorganic As in C57BL/6J mice fed five different diets containing marine fish muscle and food spiked with different proportions of AsB and inorganic As. The bioaccumulation of different As species was significantly affected by different diets. Inorganic As ingested from diets was effectively absorbed by the GI tract and then accumulated in major tissues, followed by strong oxidation, reduction, and methylation processes. AsB was readily degraded to toxic As(V) in mice, providing evidence for the biodegradation of AsB in mammals. Notably, AsB-treated mice showed higher tissue As(V) concentration than control mice following long-term exposure. This observation highlighted the potential health risk associated with long-term AsB from marine fish exposure. Therefore, taken together, our study calls for more focus on the long-term risk assessment of AsB when coastal populations consume large amounts of marine fish.

## Data Availability

Not applicable.

## Supplementary Materials

The following supporting information can be downloaded at: https://www.mdpi.com/article/10.3390/toxics11020091/s1, Figure S1: Chromatogram of AsB in Pearl gentian grouper feed. A represents the arsenic solution extracted by homogenizer and 50% methanol solution as extractant, B represents the arsenic solution microwave-assisted by microwave digestion system and 1% HNO3 as extractant; Table S1: Total As concentrations (μg/g) in stomach, intestine, heart, liver, spleen, lung, kidney, and muscle after As(III) diet, As(V) diet, Fish diet (YJ), Fish diet (ZJ), and Vehicle diet exposure. Data as mean ± SD (n = 10); Table S2: Total As, As species concentrations (µg/g) and distribution (%) in As(III) diet, As(V) diet, Fish diet (YJ), Fish diet (ZJ), and Vehicle diet. Data as mean ± SD (n = 10).
