# Supplementary material for: Significant Biotransformation of Arsenobetaine into Inorganic Arsenic in Mice
Source: Toxics. 2023 Jan 18;11(2):91. doi: 10.3390/toxics11020091 (PMC9962689; doi:10.3390/toxics11020091)
Supplement: Supplementary file 1 [file toxics-11-00091-s001.zip › toxics-2152695-supplementary.pdf]

Significant biotransformation of arsenobetaine into inorganic arsenic in mice

Jichao Zhang[1], Zijun Ye[1], Liping Huang[1], Qianyu Zhao[1], Kaige Dong[1], Wei Zhang[1]*

[1] *School of Environmental Science and Engineering, Guangzhou University, Guangzhou 510006, China*

*Corresponding author, Email: zh_wei@gzhu.edu.cn

List of tables

Figure S1. Chromatogram of AsB in Pearl gentian grouper feed. A represents the arsenic solution extracted by homogenizer and 50% methanol solution as extractant, B represents the arsenic solution microwave-assisted by microwave digestion system and 1% HNO_3 as extractant……………………………………………………………..S1

Table S1. Total As concentrations (μg/g) in stomach, intestine, heart, liver, spleen, lung, kidney, and muscle after As(III) diet, As(V) diet, Fish diet (YJ), Fish diet (ZJ), and Vehicle diet exposure. Data as mean±SD (n=10)…………………………….….......S2

Table S2. Total As, As species concentrations (μg/g) and distribution (%) in As(III) diet, As(V) diet, Fish diet (YJ), Fish diet (ZJ), and Vehicle diet. Data as mean±SD (n=10)…………...…………………………………………………………………....S3

Figure S1

Figure S1. Chromatogram of AsB in Pearl gentian grouper feed. A represents the arsenic solution extracted by homogenizer and 50% methanol solution as extractant, B represents the arsenic solution microwave-assisted by microwave digestion system and 1% HNO_3 as extractant.

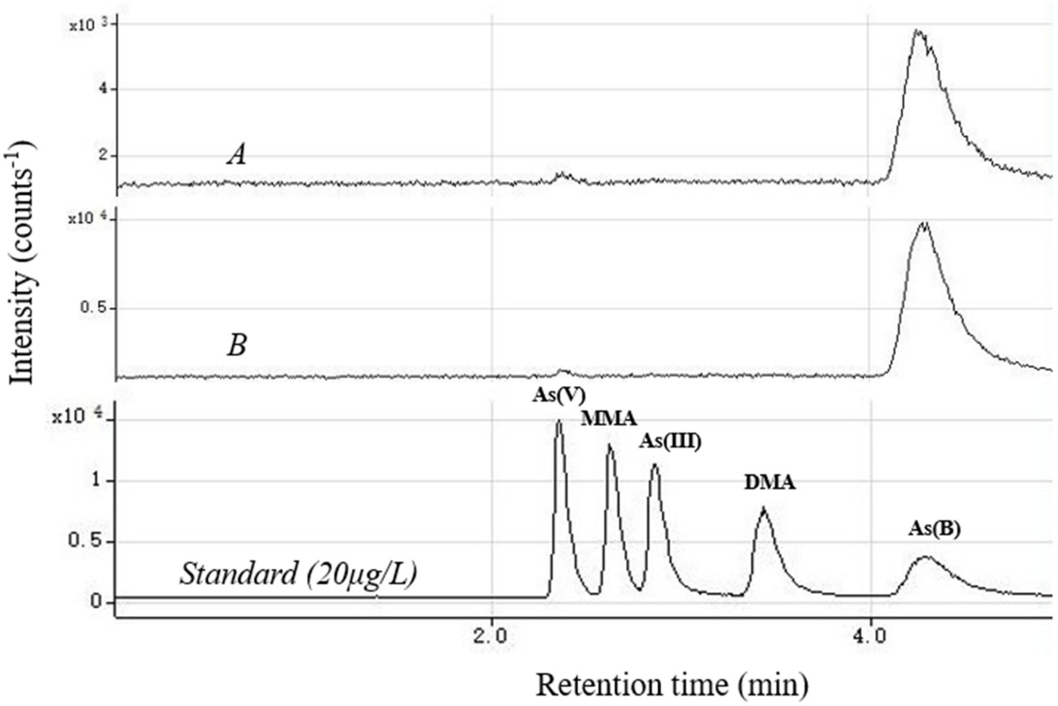

Table S1 Total As and As species concentrations (μg/g) in different tissues of mice. Below detection limit (BDL). Data as mean ± SD (n=10).

Treatment	Tissues	Arsenic species concentrations (μg/g) and distribution (%) in tissues of mice					Total As (ICP-MS) extract	Column recovery (%)
		As(III)	As(V)	MMA	DMA	AsB		
Control	Stomach	0.00450±0.000424 (55.5±2.33)	0.000200±0.0000100 (2.47±0.00125)	0.00175±0.000710 (21.7±2.01)	0.00165±0.000710 (20.4±0.190)	BDL	0.00675±0.00167	120
	Intestine	0.00287±0.000777 (35.3±13.7)	0.00230±0.00200 (24.9±17.2)	0.00137±0.000252 (16.0±0.901)	0.00200±0.000100 (23.9±3.22)	BDL	0.00832±0.00151	102
	Heart	0.000100±0.0000123 (8.28±0.0145)	0.000667±0.000208 (54.3±13.8)	0.000300±0.000100 (24.1±5.85)	0.000167±0.000115 (13.4±8.50)	BDL	0.00113±0.000191	109
	Liver	0.000958±0.000450 (23.0±9.05)	0.00107±0.000567 (26.3±11.7)	0.000740±0.000241 (18.0±3.44)	0.000620±0.000179 (15.1±1.50)	0.000680±0.000148 (17.6±5.67)	0.00396±0.00109	103
	Lung	0.00157±0.000519 (34.4±9.16)	0.000700±0.000141 (15.6±4.19)	0.00115±0.0000710 (25.5±0.167)	0.00110±0.000141 (24.5±4.80)	BDL	0.00424±0.000293	107
	Kidney	0.000300±0.0000100 (8.86±0.00347)	0.00220±0.00125 (56.7±11.1)	0.000700±0.000100 (20.0±4.34)	0.000500±0.000100 (14.4±4.24)	BDL	0.00355±0.00130	104
	Muscle	0.00117±0.000376 (39.5±8.35)	0.000250±0.000178 (9.05±4.67)	0.000650±0.000127 (22.6±5.17)	0.000840±0.000158 (28.9±3.46)	BDL	0.00271±0.000514	107
As(III) exposure	Stomach	0.240±0.00270 (68.5±0.544)	0.0121±0.000743 (3.50±0.579)	BDL	0.0981±0.0104 (28.0±0.0358)	BDL	0.338±0.0448	104
	Intestine	0.239±0.0487 (64.1±2.73)	0.0147±0.00283 (3.99±0.880)	0.0118±0.00116 (3.28±0.740)	0.106±0.0138 (28.7±2.45)	BDL	0.377±0.0687	98.6
	Heart	0.00650±0.000707 (7.93±1.64)	0.0648±0.00679 (78.2±0.448)	BDL	0.0115±0.00212 (13.8±1.19)	BDL	0.0824±0.00981	100

	Liver	0.0940±0.0110 (55.7±1.61)	0.0163±0.00769 (9.47±3.88)	BDL	0.0317±0.00519 (18.9±3.08)	0.0268±0.00212 (16.0±2.13)	0.0936±0.0119	180
	Lung	0.0470±0.00990 (35.7±0.470)	0.0426±0.00481 (32.7±2.80)	BDL	0.0420±0.0113 (31.7±2.33)	BDL	0.130±0.0253	101
	Kidney	0.0398±0.00854 (47.7±5.55)	0.0140±0.00285 (17.2±4.53)	0.0123±0.00126 (15.0±2.62)	0.0168±0.00403 (20.1±3.81)	BDL	0.0768±0.00317	108
	Muscle	0.0461±0.00646 (66.2±7.34)	0.00600±0.00283 (8.77±4.34)	BDL	0.0181±0.00911 (25.1±9.75)	BDL	0.0667±0.0124	105
As(V) exposure	Stomach	0.00450±0.00212 13.1±3.30	0.0225±0.00212 68.3±9.52	BDL	0.00200±0.00141 5.63±2.91	0.00450±0.00212 13.1±3.30	0.0330±0.00762	102
	Intestine	0.00650±0.00129 19.3±1.87	0.0178±0.00386 52.7±7.47	BDL	0.00450±0.000577 13.7±3.16	0.00475±0.00189 14.4±5.97	0.0332±0.00407	101
	Heart	0.00250±0.000577 17.6±6.55	0.0125±0.00379 82.4±6.55	BDL	BDL	BDL	0.0150±0.00331	100
	Liver	0.00586±0.000900 46.1±2.82	0.00214±0.000690 16.7±3.97	BDL	0.00114±0.000378 9.04±2.56	0.00357±0.000787 11.4±6.90	0.0139±0.00342	91.4
	Lung	0.00500±0.000100 16.4±0.00102	0.0205±0.00212 67.1±2.29	BDL	0.00300±0.00141 9.78±4.41	0.00200±0.00141 6.51±4.49	0.0287±0.0000798	106
	Kidney	0.00350±0.00100 14.1±1.67	0.0218±0.00732 85.9±1.67	BDL	BDL	BDL	0.0251±0.00806	101
	Muscle	0.00233±0.000577 12.9±1.97	0.0100±0.00173 56.0±10.8	BDL	0.00267±0.00115 14.5±4.78	0.00300±0.00100 16.6±4.81	0.0202±0.00348	89.1
Fish exposure (YJ)	Stomach	0.0290±0.00849 17.1±0.293	0.0400±0.00566 24.1±3.32	BDL	0.0115±0.00495 6.65±1.09	0.0885±0.0276 52.1±1.93	0.125±0.0559	135
	Intestine	0.0194±0.00295 7.73±1.41	0.0532±0.00813 21.1±2.69	BDL	0.00577±0.00209 2.25±0.620	0.176±0.0337 68.9±3.49	0.242±0.0323	105

	Heart	0.00900±0.00300<br>11.5±3.71	0.0213±0.00586<br>26.9±1.70	BDL	0.00500±0.00100<br>6.36±0.27	0.0437±0.0110<br>55.2±2.24	0.0673±0.0277	117
	Liver	0.0227±0.00409<br>7.39±1.18	0.0511±0.00562<br>16.7±1.81	BDL	0.00425±0.000957<br>1.38±0.282	0.228±0.0168<br>74.5±3.15	0.237±0.0549	129
	Lung	0.0142±0.001702<br>5.94±1.99	0.0405±0.00357<br>16.5±2.20	BDL	0.0152±0.00248<br>6.18±0.360	0.178±0.0503<br>71.4±4.55	0.251±0.0482	98.8
	Kidney	0.0143±0.00403<br>7.60±0.550	0.0297±0.0143<br>15.5±3.76	BDL	0.00814±0.00192<br>4.39±0.424	0.134±0.0201<br>72.6±4.36	0.181±0.0371	103
	Muscle	0.00787±0.00222<br>11.0±1.55	0.0133±0.00377<br>18.6±2.69	BDL	0.00805±0.00165<br>11.6±2.47	0.0414±0.00653<br>58.9±2.11	0.0725±0.0155	97.4
Fish exposure (ZJ)	Stomach	0.0183±0.00327<br>16.1±4.34	0.0396±0.00764<br>34.2±3.42	BDL	0.0219±0.00157<br>19.0±0.422	0.0355±0.00487<br>30.8±1.34	0.0976±0.0415	118
	Intestine	0.0195±0.00683<br>11.2±2.00	0.0360±0.00983<br>21.1±5.51	BDL	0.0207±0.00390<br>12.1±1.63	0.0945±0.0130<br>55.6±5.55	0.172±0.0223	99.2
	Heart	0.0144±0.000635<br>17.8±1.28	0.0320±0.00283<br>39.2±1.09	BDL	0.0143±0.00329<br>17.3±1.90	0.0210±0.00283<br>25.7±0.481	0.0794±0.0126	103
	Liver	0.0157±0.00308<br>7.60±2.58	0.0316±0.0107<br>14.4±3.46	BDL	0.0144±0.00267<br>6.67±0.536	0.156±0.0416<br>71.3±4.51	0.182±0.0556	120
	Lung	BDL	0.0432±0.00445<br>21.5±0.315	BDL	0.0304±0.00226<br>15.2±0.662	0.127±0.0169<br>63.3±0.977	0.200±0.0211	100
	Kidney	0.0169±0.00550<br>13.0±0.662	0.0380±0.0113<br>29.3±0.651	BDL	0.0153±0.00464<br>11.8±0.33	0.0591±0.0143<br>46.0±1.64	0.145±0.0520	89.2
	Muscle	0.00765±0.00411<br>12.3±7.27	0.0133±0.00390<br>21.1±5.80	BDL	0.0181±0.00503<br>28.6±5.49	0.0237±0.00430<br>38.0±7.21	0.0652±0.00971	96.2

Table S2 Total As, As species concentrations (µg/g) and distribution (%) in As(III) diet, As(V) diet, Fish diet (YJ), Fish diet (ZJ), and Control diet. S1
Data as mean ± SD (n = 10).

	Arsenic species concentrations (µg/g) and distribution (%) in diets					Total As (µg/g) (ICP-MS extract)	Column recovery (%)
Treatments	As(III)	As(V)	MMA	DMA	AsB		
Control diet	0.00229±0.000126<br>(8.29±0.688)	0.0162±0.00229<br>(58.0±3.51)	BDL<br>BDL	0.00101±0.000166<br>(3.63±0.534)	0.00837±0.00160<br>(30.0±3.42)	0.0289±0.00154	93.1
As(III) diet	0.843±0.0356<br>(78.6±1.21)	0.190±0.0119<br>(17.8±1.08)	BDL<br>BDL	BDL<br>BDL	0.0387±0.00154<br>(3.64±0.401)	1.09±0.0611	98.3
As(V) diet	0.0263±0.000187<br>(9.29±1.12)	0.191±0.0391<br>(66.6±5.65)	BDL<br>BDL	0.00542±0.000640<br>(1.94±0.463)	0.0626±0.00714<br>(22.2±4.16)	0.301±0.0213	94.7
Fish diet (YJ)	0.00719±0.000329<br>(0.497±0.0760)	0.0592±0.00739<br>(4.10±0.370)	BDL<br>BDL	0.0133±0.00109<br>(0.925±0.105)	1.36±0.0405<br>(94.5±0.342)	1.44±0.0486	99.9
Fish diet (ZJ)	0.0317±0.00368<br>(2.76±0.496)	0.0762±0.00463<br>(6.70±1.10)	0.0645±0.0253<br>(5.76±2.77)	0.0287±0.00510<br>(2.50±0.411)	0.950±0.147<br>(82.3±4.29)	1.26±0.0455	91.4

Values are means ± SD (n=10), BDL means below detection limit.
